# Emergency Department-Centered Digital Health Transformation: Narrative Review and Three-Pillar Implementation Framework

**DOI:** 10.5811/westjem.66747

**Published:** 2026-10-27

**Authors:** Sarah Sossong, Aline Kalaydjian, Sarah Pletcher, Christopher Ziebell, Ngoc-Anh A. Nguyen

**Affiliations:** * Houston Methodist Hospital, Center for Connected Care Innovation and Implementation-Research, Houston, Texas; † Houston Methodist Hospital, Houston Methodist Research Institute, Houston, Texas; ‡ Houston Methodist Hospital, Department of Surgery, Houston, Texas; § Houston Methodist Hospital, Department of Medicine, Houston, Texas; || US Acute Care Solutions, Canton, Ohio

## Abstract

**Introduction:**

Emergency departments (EDs) face escalating pressures from rising patient volumes, workforce shortages, and documentation burdens. Digital health technologies have shown promise individually, but most are implemented in isolation, limiting their collective impact. This narrative review synthesizes evidence for six digital health technologies applicable to the ED and proposes a three-pillar framework for their coordinated implementation, positioning the ED as a convergence point for health system digital transformation.

**Methods:**

We searched PubMed and Google Scholar for peer-reviewed literature published from 2018 through July 2026 on ambient artificial intelligence (AI) documentation, telehospitalist programs, virtual nursing, remote patient monitoring, virtual specialty consultation, and postdischarge transitional care, prioritizing systematic reviews and meta-analyses. Fifty-one sources met inclusion criteria and were synthesized. We organized the technologies into three functional pillars: streamlining clinical workflows; expanding workforce capacity; and extending continuity of care. Outcomes included clinical, operational, and implementation measures. Experience from Houston Methodist, a nine-hospital health system, is presented as an illustrative example separate from the literature synthesis.

**Results:**

Of the 51 sources, 23 (45.1%) addressed streamlining clinical workflows, 16 (31.4%) expanding workforce capacity, and 12 (23.5%) extending continuity of care. Overall, 22 (43.1%) were ED-specific: 10, five, and seven within the respective pillars. Ambient AI documentation included 13 sources (seven ED-specific); telestroke included one ED-specific source. Evidence maturity varied by technology and setting. In non-ED settings, meta-analyses reported shorter documentation time with ambient AI documentation (standardized mean difference, −0.72; 95% CI, −0.99 to −0.45), lower hospital mortality with tele-intensive care (risk ratio, 0.74; 95% CI, 0.58–0.96), and lower odds of 30-day readmission with low-complexity post-discharge interventions (odds ratio [OR], 0.78; 95% CI, 0.66–0.92). Emergency department-specific evidence remained largely observational; one meta-analysis showed no mortality difference with telestroke (OR, 1.16; 95% CI, 0.94–1.43), while one systematic review found no demonstrable benefits from telephone follow-up post-ED discharge.

**Conclusion:**

Current evidence supports individual digital health technologies in and around the ED. Based on implementation science principles and institutional experience, we propose that coordinated implementation across the three pillars may offer advantages over isolated deployments, a hypothesis warranting prospective comparative evaluation.

## INTRODUCTION

Emergency departments (EDs) occupy a unique position at the intersection of acute care delivery and health system operations, serving as both safety nets for urgent medical needs and potential launch points for comprehensive care coordination. Mounting operational pressures contribute to prolonged boarding, delayed specialist access, and fragmented postdischarge follow-up. These challenges have important consequences for community health outcomes, disproportionately affecting patients with limited access to primary care who depend on the ED as their principal point of contact with the health system. Coordinated digital transformation may offer a systems-level response, improving care delivery for individual patients and the broader communities EDs serve.

Emergency medicine (EM) continues to experience substantial workforce strain, including workforce shortages,^1^ documentation burden,^2^ and clinician well-being concerns.^3^ Burnout remains high among emergency physicians, with rates approaching 60%,^4^ reflecting cumulative pressures from clinical demands, shift work, documentation burdens, and administrative complexity.^4^,^5^ Studies project a growing shortfall in physician supply concurrent with increasing care demands from aging populations.^6^–^8^ Effective solutions require coordinated interventions across workflow optimization, technology integration, and organizational support, because clinician well-being directly impacts patient safety and care quality.^9^

The COVID-19 pandemic demonstrated rapid adoption of virtual care technologies, dismantling long-standing barriers to telemedicine integration,^10^ and illustrating how digital tools can enhance, rather than burden, clinical workflows, including in the ED.^11^ Patient acceptance of telehealth has grown in parallel, with improved convenience, broader access, and generally positive experiences.^12^ Emerging studies further highlight the potential of digital health technologies, including large language models and ambient artificial intelligence (AI), to improve documentation quality, reduce administrative burden, and enhance clinician satisfaction in emergency settings.^13^–^15^

Despite growing interest in digital health solutions for EM,^16^ prior ED-specific literature has predominantly evaluated individual technologies or narrowly defined applications, including ambient documentation,^17^,^18^ broader AI applications,^19^–^21^ digital decision tools,^22^,^23^ and telemedicine.^24^–^30^ Broader evidence syntheses have examined digital transformation at the hospital or health-system level, with limited focus on ED-specific implementation.^31^–^33^ Coordinated implementation of multiple digital technologies in the ED remains largely unexplored. The present review extends this literature by proposing the ED as a convergence point where multiple technologies can be integrated through a coordinated implementation framework.

We address three specific aims: (1) to synthesize current evidence on key digital health technologies applicable to ED settings, including ambient AI documentation, virtual nursing, telehospitalists, remote patient monitoring, virtual specialty consultation, and postdischarge transitional care; (2) to describe implementation frameworks and success factors for coordinated digital transformation within EDs; and (3) to identify evidence gaps and future research priorities for advancing this field. To complement the published literature with illustrative evidence, we present implementation experience from Houston Methodist, a nonprofit health system with nine hospitals, including a flagship academic medical center in the Texas Medical Center.

## METHODS

We identified relevant literature through targeted searches of PubMed and Google Scholar covering peer-reviewed literature published from 2018 through July 2026. Searches combined keywords related to emergency medicine (such as “emergency department” and “emergency medicine”) and digital health technologies (such as “digital health,” “digital transformation,” “ambient AI,” “telehospitalist,” “virtual nursing,” “remote patient monitoring,” and “transitional care intervention”), using Boolean (AND/OR) operators. Sources were included if they evaluated digital health technologies, were conducted in the ED setting or had potential applicability to the ED, and were peer-reviewed original research, systematic reviews, meta-analyses, scoping reviews, and implementation studies or reports. We excluded sources if they evaluated digital health technologies without applicability to the ED or were conference abstracts without full-text availability. Two members of the study team reviewed identified sources against the inclusion and exclusion criteria, with eligibility and inclusion decisions made collaboratively through discussion. When available, systematic reviews and meta-analyses were prioritized over individual primary studies to synthesize the evidence. Reference lists of identified articles were reviewed to identify additional relevant publications.

Of 125 sources reviewed, we included 51. Included sources were categorized by digital health technology and synthesized according to clinical setting (ED vs non-ED) and reported outcome domain, including clinical, operational, and implementation outcomes. We chose this narrative approach over systematic review due to the heterogeneous nature of interventions and outcomes across studies, which precluded meaningful meta-analysis. Institutional implementation experience, developed through iterative Plan-Do-Study-Act cycles, was incorporated as an illustrative case example and was not considered part of the literature synthesis. Because these activities were conducted as operational quality improvement initiatives, they did not constitute human subjects research requiring institutional review board approval.

## RESULTS

### Digital Health Technologies in Emergency Medicine

Health systems nationwide have implemented diverse interventions to address documentation efficiency, clinician well-being, workforce capacity, and continuity of care.^9^,^15^ We organized the evidence around three functional pillars: streamlining clinical workflows; expanding workforce capacity; and extending continuity of care (Figure 1).

Of the 51 included sources, 23 (45.1%) addressed streamlining clinical workflows, 16 (31.4%) expanding workforce capacity, and 12 (23.5%) extending continuity of care. Overall, 22 (43.1%) sources were ED-specific: 10, 5, and 7 within the respective pillars. Ambient AI documentation was the most represented technology with 13 sources, including seven ED-specific sources, while telestroke included one ED-specific source. Table 1 summarizes the evidence base, key outcomes, and limitations for each technology reviewed.

### Pillar 1: Streamlining Clinical Workflows

#### Ambient Artificial Intelligence Documentation

Ambient AI documentation systems passively capture clinician-patient conversations and generate structured, electronic health record (EHR)-compatible notes. Recent systematic reviews of AI-powered documentation reported shorter documentation time (standardized mean difference [SMD], −0.72; 95% CI, −0.99 to −0.45),^34^ particularly for complex cases,^35^ but emphasized the need for continued evaluation of patient outcomes^34^ and documentation quality,^34^,^35^ given hallucinated^37^ and omitted content.^35^ Adoption was also associated with reduced work exhaustion (mean difference [MD], −0.44 points; 95% CI, −0.62 to −0.25).^36^ In a randomized trial, both DAX and Nabla AI scribes improved well-being (Mini-Z score differences, 2.83 [95% CI, 1.28–4.37] and 2.69 [95% CI, 1.14–4.23], respectively); DAX also reduced task load (MD, −39.9; 95% CI, −71.9 to −7.9).^37^ A multisite study showed 13.4 fewer EHR minutes per eight scheduled patient-hours (95% CI, 9.1–17.7) and 0.49 additional weekly visits (95% CI, 0.17–0.81).^38^ Large-scale implementation of more than 2.5 million uses highlighted continued improvements in clinician satisfaction and workflow efficiency.^15^

Emergency department-specific evidence remains comparatively limited, reflecting recent adoption. In a retrospective study, ambient AI use was associated with shorter median on-shift documentation time (165 [interquartile range (IQR), 97–250] vs 230 [IQR, 142–365] seconds; 28% reduction) and total EHR time (519 [IQR, 349–751] vs 621 [IQR, 412–914] seconds; 16% reduction).^39^ In a simulation study, AI note editing was faster than dictation (75 vs 125 seconds; 39% reduction; *P* < .001).^40^ Documentation generated by large language models was generally accurate, although errors and clinically relevant omissions remained.^41^ Emergency physicians generally viewed AI and ambient documentation positively,^42^,^43^ with 71.4% reporting improved documentation efficiency (95% CI, 45.4%–88.3%)^44^ and participants in a crossover evaluation reporting preserved note quality.^45^

#### Telehospitalist Integration

Telehospitalist programs provide remote access to hospitalist expertise. Across inpatient settings, studies demonstrated feasibility,^46^–^48^ scalability,^48^,^49^ and sustainability.^50^ Integration reduced outside transfers from the ED (16.6% vs 10.5%; *P* < .001) and transfers at any time (17.3% vs 11.9%; *P* < .001).^51^ A retrospective comparison with traditional locum hospitalist care found no significant differences in length of stay (LOS) (mean [standard deviation (SD)], 2.82 [2.49] vs 2.55 [3.06] days; *P* = .14), in-hospital mortality (1.7% vs 2.3%; *P* = .51), or 30-day readmissions (1.83% vs 2.28%; *P* = .61).^52^ Overall, patients^46^,^52^ and staff^46^,^51^ reported high satisfaction.

Evidence evaluating telehospitalist integration in the ED remains comparatively limited. Early evaluation of a nocturnal program designed to reduce on-site hospitalist coverage reported positive patient and care-team experience.^53^ A more recent nocturnal program centralizing overnight ED admissions demonstrated implementation feasibility across a multisite health system.^54^ Another program supporting ED clinical decision-making was associated with optimized hospital bed utilization and facilitated safe discharges without negative effects on patient outcomes.^55^

### Pillar 2: Expanding Workforce Capacity

#### Virtual Nursing

Virtual nursing is designed to interact with patients using secure video connections, allowing bedside nurses to focus on direct patient care.^56^ A scoping review reported that virtual nursing in the inpatient setting primarily supports admissions, discharges, care coordination, and nursing workflows; patient and nurse experiences were generally positive.^57^ A retrospective evaluation showed no significant differences from usual care in intensive care unit (ICU) transfer (average marginal effect [AME], −0.08 percentage points; 95% CI, −1.34 to 1.18), inpatient mortality (AME, 0.43 percentage points; 95% CI, −0.33 to 1.18), or LOS (AME, −0.20 days; 95% CI, −0.57 to 0.17).^58^ Recurrent implementation themes included clearly defined virtual nursing roles,^56^,^59^ bedside nurse engagement during model development,^60^,^61^ and virtual workflow education.^59^

Published ED-specific evidence remains scarce. One multicenter retrospective study associated virtual nursing with shorter median wait times (429.5 [IQR, 292.6–954.5] vs 525.5 [IQR, 332.9–969.8] minutes; *P* < .001), fewer admission-assessment interruptions (mean [SD], 1.4 [1.3] vs 1.8 [1.5]; *P* < .001), and fewer incomplete documentation fields (0.2 [0.5] vs 0.4 [1.5]; *P* < .001) compared with standard in-person admission assessments, suggesting a promising care-delivery model in the ED.^62^

#### Continuous Remote Patient Monitoring

Wearable remote patient monitoring devices continuously track physiologic parameters and can integrate EHR workflows and early warning systems.^63^,^64^ An early inpatient systematic review supported deployment feasibility and measurement validity.^65^ Phased implementation across an eight-hospital system further supported scalability.^63^ Systematic reviews and meta-analyses found no significant differences compared with traditional monitoring in major events or complications (odds ratio [OR], 0.71; 95% CI, 0.38–1.31), ICU admission (OR, 0.82; 95% CI, 0.25–2.67),^66^ rapid response activation (risk ratio [RR], 0.61; 95% CI, 0.26–1.43),^67^ or LOS (MD, −0.09 days; 95% CI, −0.46 to 0.28).^68^ Mortality findings varied; one meta-analysis reported lower mortality (RR, 0.61; 95% CI, 0.39–0.95)^67^ whereas others did not.^66^,^68^

Emergency department evidence for remote patient monitoring remains limited to feasibility, validation, and predictive modeling studies. Deployment was feasible and acceptable to patients.^69^ Among patients with suspected sepsis, device-derived heart rate (*r* = 0.87, *P* < .001) and respiratory rate (*r* = 0.75, *P* < .001) correlated strongly with manual measurements.^70^ A wearable device detected deterioration earlier than standard monitoring (MD, 39.48 minutes; *P* < .001),^71^ while models incorporating continuous physiologic data predicted septic shock earlier than those using manually collected data.^72^

### Pillar 3: Extending Continuity of Care

#### Virtual Specialty Consultation

Virtual specialty consultations expand timely access to subspecialist care. In inpatient settings, strong evidence supports tele-ICU integration, while evaluations of telepsychiatry programs are emerging. A tele-ICU meta-analysis reported lower ICU mortality (RR, 0.83; 95% CI, 0.72–0.96), hospital mortality (RR, 0.74; 95% CI, 0.58–0.96), and ICU LOS (MD, −0.63; 95% CI, −1.09 to −0.17), although substantial heterogeneity across models was highlighted.^73^ A systematic review of inpatient telepsychiatry reported implementation feasibility across diverse US general hospitals.^74^

Evidence evaluating virtual specialty consultations in the ED varies by specialty. Telestroke, designed for the acute management of stroke patients, is particularly applicable to the ED. A meta-analysis found no significant differences with conventional in-person stroke care in onset-to-intravenous tissue plasminogen activator times (mean [SD], 144.09 [18.87] vs 147.18 [25.97] minutes; *P* = .63), door-to-needle times (73.03 [20.04] vs 65.91 [25.96] minutes; *P* = .32), or 90-day functional independence (modified Rankin Scale score of 0–2; OR, 1.06; 95% CI, 0.89–1.29), mortality (OR, 1.16; 95% CI, 0.94–1.43), or symptomatic intracranial hemorrhage (OR, 0.99; 95% CI, 0.73–1.34).^75^ Tele-ICU was associated with lower in-hospital mortality among those admitted to the ICU (adjusted OR, 0.20; 95% CI, 0.06–0.70).^76^ Telepsychiatry was generally feasible and acceptable to stakeholders, enabling on-demand consultations that may reduce waiting times for psychiatric assessment, although evidence regarding clinical effectiveness remains limited.^77^

#### Postdischarge Transitional Care

Systematic reviews and meta-analyses have evaluated heterogeneous posthospital care interventions, including telephone-based follow-up, home visits, and telemonitoring. Compared with usual care, discharge communication interventions were associated with fewer 30-day readmissions (RR, 0.69; 95% CI, 0.56–0.84) and greater treatment adherence (RR, 1.24; 95% CI, 1.13–1.37).^78^ Nurse-led interventions showed fewer readmissions with follow-up longer than 12 weeks (RR, 0.67; 95% CI, 0.49–0.92) and fewer ED visits (RR, 0.63; 95% CI, 0.49–0.81).^79^ Effects varied by complexity: low-complexity interventions were associated with lower odds of readmission at 30 days (OR, 0.78; 95% CI, 0.66–0.92) and 180 days (OR, 0.45; 95% CI, 0.30–0.66); medium-complexity interventions, at 90 days (OR, 0.64; 95% CI, 0.45–0.92); and high-complexity interventions, with shorter LOS (SMD, −0.20; 95% CI, −0.38 to −0.03) and greater patient satisfaction (SMD, 0.52; 95% CI, 0.22–0.82).^80^

Evidence supporting transitional care interventions post-ED discharge is emerging but remains less mature than following inpatient discharge. Comprehension-focused interventions were the most studied and generally improved patient understanding, although heterogeneous designs limit generalizability.^81^ Intervention effectiveness varied by modality and structure. Nurse-led telephone follow-up did not decrease readmissions and revisits,^82^ whereas structured telehealth was associated with lower odds of revisit at 7 days (OR, 0.69; *P* < .001) and 30 days (OR, 0.82; *P* < .001).^83^ Noncompletion of a structured transitional care visit was associated with higher odds of 90-day ED revisit (OR, 2.11; 95% CI, 1.02–4.33). Among completers, 26% were referred back to the ED and most were subsequently admitted, which the authors interpreted as appropriate identification and escalation of clinical deterioration.^84^ Structured follow-up may hold particular value for uninsured and underinsured patients, for whom the ED is often the only health-system contact.

## DISCUSSION

### Coordinated Implementation Frameworks

Several reviews have examined individual digital health technologies relevant to EM, including ambient AI documentation,^34^,^35^ telestroke,^75^ tele-ICU,^73^ telepsychiatry,^77^ virtual nursing,^57^ remote patient monitoring,^65^,^68^ and ED discharge interventions.^81^ Collectively, these reviews evaluated feasibility, implementation, operational performance, and clinical outcomes, but each focused on a single technology. The present review synthesizes evidence across six technologies, positions the ED as a convergence point for coordinated digital transformation, and proposes a three-pillar framework for implementation.

While individual technologies demonstrate promise, coordinated implementation frameworks may offer advantages by enabling workflow integration and reducing duplicative efforts. Implementation science frameworks highlight that successful digital transformation depends on the integration of technologies within organizational and clinical systems rather than isolated deployments.^85^ Evidence from broader digital hospital transformations suggests that integrated, system-level implementation of interoperable technologies is associated with improvements in both patient and clinician experience, reinforcing the importance of coordinated approaches over isolated deployment.^88^ Figure 2 illustrates how these technologies may activate chronologically across the patient journey: virtual nursing intake and ambient AI documentation on arrival; virtual specialty consultation if indicated; remote patient monitoring; telehospitalist admission during boarding; and virtual discharge education with structured follow-up after discharge. This coordination may facilitate data flow and longitudinal care across the acute and postacute continuum rather than siloed deployments.

### Critical Implementation Factors

Successful coordinated digital transformation relies on key factors highlighted in recent implementation reports, including governance, project management, implementation science and change management best practices, workflow integration, and clinician oversight.^89^ These factors are not unique to any single technology and represent foundational elements of digital transformation.

#### Governance and Data Protection

Effective implementation of digital health technologies demands strong governance, privacy safeguards, and appropriate clinician oversight. The World Health Organization emphasizes that telemedicine implementations must ensure patient safety, privacy, traceability, accountability, and security across all digital modalities.^86^ Similar principles apply to AI-based documentation tools, where strong data governance and clinician oversight remain essential, as demonstrated in applied implementations of clinical AI systems.^87^ Health system innovation leadership must work closely with information technology (IT) security, compliance, and clinical stakeholders to ensure data protection across technology types.

#### Electronic Health Record Integration

Embedding tools within existing EHR workflows reduces workflow disruption and training burden, which may increase adoption,^85^,^88^,^89^ whereas technologies requiring separate systems or additional login credentials may create significant barriers, particularly in high-pressure ED environments.^85^,^90^

#### Stakeholder Engagement and Iterative Improvement

Early and continuous involvement of frontline staff is essential for successful adoption and workflow integration.^85^,^91^ Clinician participation in design, testing, and refinement supports sustained use, and training should focus on workflow integration rather than technology operation alone.^85^,^89^ Plan-Do-Study-Act cycles provide a structured approach to iterative testing, feedback, and staged refinement of implementation strategies.^92^

#### Adoption Barriers

Evidence from implementation reports, observational studies, and systematic reviews suggests that technology adoption is influenced by clinician, technology, and organizational factors. Clinician trust in digital tool outputs remains divided. While ambient AI may increase documentation quality, fictitious information or “hallucinations” in AI-driven documentation, in addition to misplaced information, unnecessary wordiness, and omissions raise concerns regarding the reliability and accuracy of these technologies.^35^,^36^,^93^ Uncertainty around responsibility and liability for inaccuracies in documentation may further limit implementation in clinical settings.^93^ Health technologies may also increase cognitive load by requiring clinicians to adapt to new workflows and manage technology-related disruptions.^90^

Technological challenges, including network instability, hardware malfunctions, insufficient specialty-specific customization, and data security concerns, may disrupt workflows and hinder use, requiring technical support and continued training to maintain digital competency.^90^,^93^,^94^

At the organizational level, skepticism toward new technologies and change fatigue, particularly when multiple technologies are implemented and refined concurrently, may further hinder adoption.^90^

### Illustrative Implementation Experience from Houston Methodist

Houston Methodist is a nonprofit nine-hospital health system, including a flagship quaternary academic medical center in the Texas Medical Center. Between 2022 and 2025, multiple digital health technologies were deployed sequentially through coordinated quality improvement initiatives across hospital sites. Key infrastructure supporting coordinated implementation included an existing system-wide Epic EHR platform, a centralized 24/7 Virtual Operations Center (VOC), strong IT support, and innovation leadership. Focus groups, regular surveys, and usage analytics guided iterative refinement, and the ED, with its rapid turnaround, frequent handoffs, and multidisciplinary workflows, served as a testing ground for coordinated digital health innovation. The examples presented below are intended to be illustrative of coordinated digital transformation; they do not establish causal effectiveness.

#### Ambient Artificial Intelligence Documentation

An ambient AI documentation platform (Ambience Healthcare) was integrated into Epic through a staged rollout, from 10 emergency physicians to over 150 users system-wide. Quality improvement data demonstrated reduction in documentation time, early signals for improved coding accuracy, and low rates for elective discontinuation. Mitigation strategies for hallucinations included mandatory clinician attestation, targeted error audits, and rapid vendor feedback loops.

#### Telehospitalist Integration

The telehospitalist model for nocturnal inpatient admissions from the ED was designed to alleviate boarding, staffing shortages, and challenges associated with increasing patient complexity. Implementation occurred sequentially across four sites and was led by a multidisciplinary team comprising physicians and operations leaders. The team developed standardized workflows for consultation initiation, documentation, and staff training, facilitating consistent admission coverage across sites.

#### Virtual Nursing

Virtual nurses conduct standardized intake assessments including safety screening, allergy verification, and psychological assessments. In the ED, virtual sitters support select patients requiring continuous observation, such as those with psychiatric conditions. Engaging bedside nurses in the design phase ensured virtual nursing complemented rather than replaced direct care.

#### Continuous Remote Patient Monitoring

The BioButton (BioIntelliSense Inc) wearable remote patient monitoring device was deployed across all hospitals, with continuous physiologic data transmitted hourly to Epic for early warning score integration. The VOC team monitors approximately 2,000 to 3,000 patients system-wide; triggered alerts escalate from technicians to virtual intensive care unit (VICU) nurses, who may activate the rapid response team. In the ED, the BioButton supported monitoring of patients awaiting admission, where nursing ratios may limit traditional vital sign frequency.

#### Virtual Specialty Consultation

Telestroke, telepsychiatry, VICU, and telepharmacy services are fully integrated into Epic workflows and standardized across all nine hospitals. The VICU model exemplifies multidimensional coordinated implementation: it extends continuity of care from the ED to the ICU, delivers 24/7 coverage without additional on-site staffing, and accelerates critical care transition planning.

#### Postdischarge Transitional Care

Existing virtual care infrastructure and staffing models were leveraged to implement a post-ED discharge transitional care program, requiring minimal additional operational resources and minor workflow changes. Automated text messages invited patients to schedule nurse practitioner-led virtual follow-up visits focused on medication adherence, symptom monitoring, and care plan reinforcement.

### Implementation Considerations for Health Systems

Successful implementation of coordinated digital transformation frameworks requires several prerequisites: robust IT infrastructure and support; strong governance and change management capabilities; established virtual care capabilities; and leadership commitment to a coordinated approach. These contextual factors were essential to the digital transformation at Houston Methodist.

Successful implementation of all six technologies remains challenging, particularly for resource-limited systems. Prioritization should consider evidence maturity, expected clinical and operational value, infrastructure requirements, and local needs. Table 2 presents a proposed prioritization, classifying each technology as foundational (must-have) or enhancing (nice-to-have) alongside the infrastructure burden associated with initial implementation.

The three pillars are functional groupings rather than an implementation sequence, and prioritization cuts across them. Technologies extending continuity of care emerged as the highest priority for resource-limited settings, followed by workflow technologies and then workforce technologies, reflecting differences in infrastructure burden and evidence maturity rather than the order in which the pillars are presented.

The coordinated digital transformation framework adapts to different hospital sizes, supports phased implementation that reduces resource burden, and allows local customization.

Integration into standard workflows, reallocation of staff time, clinician satisfaction, and training all support sustainability.

Although formal cost-effectiveness analyses remain limited, most digital health technologies have demonstrated operational improvements that may translate into economic benefits. Ambient AI documentation has been associated with improved clinician productivity and visit volumes,^38^ while virtual nursing programs may reduce labor costs.^57^ By decreasing readmissions and ED revisits,^83^ transitional care interventions may further decrease costs related to health care utilization. Similarly, virtual specialty consultations such as tele-ICUs may reduce costs associated with ICU utilization.^95^ Beyond the potential value of individual technologies, a coordinated framework may offer additional opportunities, including leveraging shared infrastructures, centralized virtual clinicians, EHR integration workflows, and common governance structures.

Equity considerations are central to the population health mission of EM. Digital tools may offer opportunities to address unmet social needs and promote more equitable care.^22^ However, concentrating digital transformation in well-resourced systems risks widening disparities in community health outcomes by limiting technology-enabled care among the patients who stand to benefit most.^96^–^99^ Safety-net and rural EDs face barriers: digital tools require upfront investment, technical infrastructure, and dedicated resources that may be beyond the reach of systems already managing workforce shortages and limited broadband access. Partnerships, shared resources, and policy initiatives addressing implementation barriers may be necessary to promote equitable technology adoption across diverse health care settings.^100^,^101^

### Future Research Directions

Future research priorities should include longer-term outcomes assessment, cost-effectiveness analyses, and formal pre- and postimplementation studies with control groups to strengthen the evidence base.^50^ Additional opportunities include extending implementation frameworks and toolkits beyond EM^85^ and integrating AI for predictive analytics and clinical decision support, where large language models have demonstrated promising ED triage performance in controlled comparative studies.^102^,^103^

Multisite randomized or quasi-experimental studies across health system types would help establish causality and generalizability. Critically, comparisons of coordinated multi-technology implementation against isolated deployments would determine whether the synergies described in this review, such as data continuity across care transitions and reduced workflow fragmentation, translate into measurable clinical and operational differences.

These priorities point toward expansion of remote patient monitoring into the home as a key next step, strengthening longitudinal connections across hospital, home, and clinic settings.^104^ Hospital-at-home models, which combine remote physiologic monitoring, in-home nursing, and virtual physician oversight, represent an adjacent frontier.^105^

## LIMITATIONS

The evidence base for digital transformation in EM is rapidly evolving but uneven in maturity (Table 1). Systematic reviews and meta-analyses support established technologies such as telestroke and tele-ICU, and have now emerged for ambient AI documentation,^34^,^35^ including early randomized trials.^36^,^37^ In contrast, evidence for newer innovations such as virtual nursing remains largely descriptive, and ED-specific evidence across technologies is predominantly observational. Randomized controlled trials remain rare overall, limiting causal attribution. Notably, this review did not identify comparative studies evaluating coordinated versus isolated technology implementations, limiting the ability to attribute observed outcomes specifically to the coordinated approach rather than to individual technologies.

Generalizability may also be limited by setting. Financial pressures, competing priorities, and limited technical capacity present implementation barriers across health systems of all types. Additionally, selection bias may affect reported outcomes, particularly for programs like virtual follow-up where patient participation is voluntary.

The narrative review process itself has several limitations. While appropriate for synthesizing heterogeneous evidence, narrative reviews do not provide the methodological rigor of systematic reviews or meta-analyses, limiting their ability to quantify associations or establish causation. Significant literature may be missed, and interpretation of evidence remains author and context dependent; in this review, interpretation may additionally be influenced by the authors’ institutional experience, which we mitigated by positioning the Houston Methodist experience as an illustrative case, excluded from the literature synthesis, that reflects a single health system and may not generalize to other settings. The rapid pace of technological development means newer implementations and evidence may emerge quickly after publication. Despite these limitations, the literature offers real-world implementation data from multiple institutions, growing sample sizes as technologies scale, consistent findings across settings, and clinically meaningful endpoints beyond simple adoption metrics.

## CONCLUSION

The intersection of rising patient acuity, frequent handoffs, persistent boarding, and unpredictable staffing variability uniquely positions EDs as convergence points for coordinated digital transformation. Current evidence, supplemented by implementation experience from Houston Methodist, suggests that coordinated approaches may better integrate digital tools into ED workflows than isolated deployments. A three-pillar framework addressing workflow optimization, workforce capacity expansion, and continuity of care proposes a systematic approach for health systems seeking comprehensive digital transformation. Key implementation factors include stakeholder engagement, EHR integration, and iterative improvement cycles. Success should be measured not just by operational metrics but by improved access to timely care, higher clinician satisfaction, and stronger capacity to serve all patients effectively. Realizing the full population health potential of this transformation will require deliberate policy and partnership strategies that extend these benefits equitably to the communities and patients who need them most, including those for whom the ED remains the primary point of access to the health system.

## Figures and Tables

**Figure 1:**
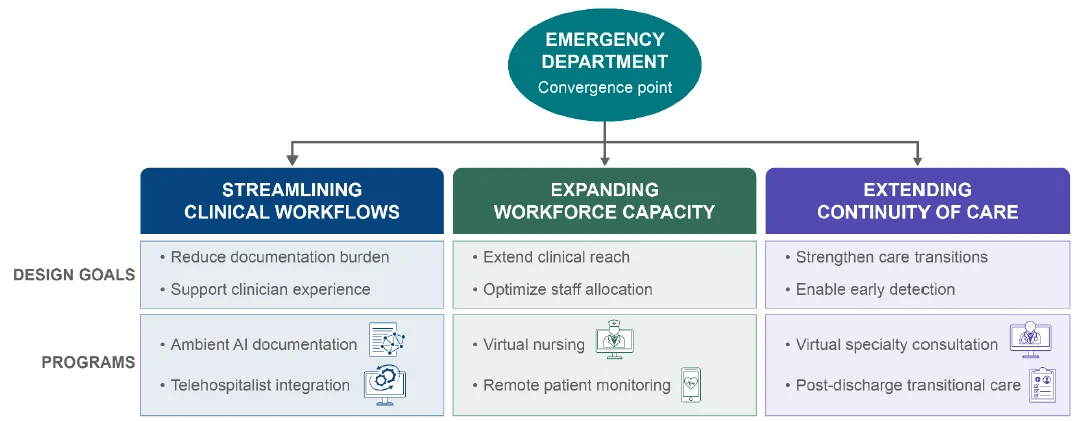
The emergency department as a convergence point for coordinated digital health transformation: a three-pillar framework. *AI*, artificial intelligence.

**Figure 2:**
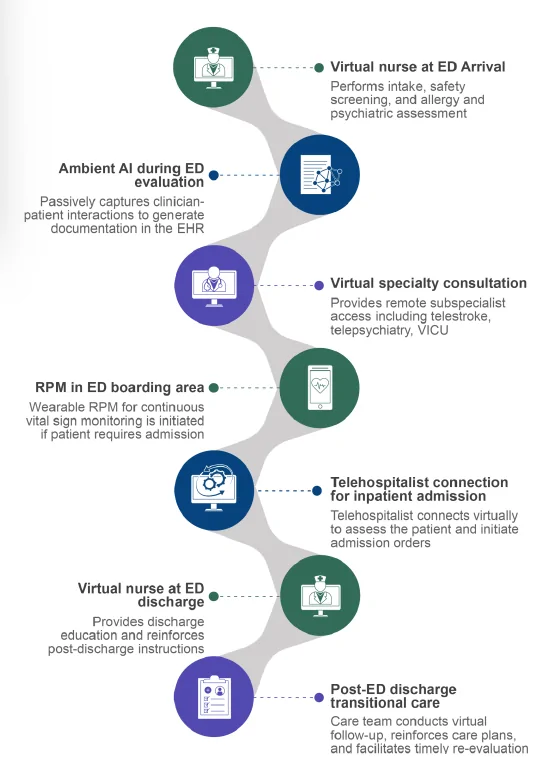
Digital health technology activation across emergency department care phases: from arrival through postdischarge follow-up. *AI*, artificial intelligence; *ED*, emergency department; *EHR*, electronic health record; *RPM*, remote patient monitoring; *VICU*, virtual intensive care unit.

**Table 1:** Summary of evidence for digital health technologies in non-emergency department (ED) and ED settings in a review synthesizing evidence for six digital health technologies applicable to the ED.

Three-Pillar Framework	Technology	ED Use Case	Evidence in Non-ED Settings	Evidence in ED Settings	Key Limitations
Pillar 1: Streamlining Clinical Workflows (*n* = 23; ED = 10)	Ambient AI documentation (*n* = 13; ED = 7)	Automated note generation from clinician-patient conversations	SR/MA: reduced documentation time (Zhao 2026; Bracken 2025) RCT: improved well-being and reduced task load (Lukac 2025). Reduced work exhaustion (Afshar 2025) Observational: decreased EHR time, increased weekly visit volume (Rotenstein 2026) Implementation studies: >2.5M uses with improved clinician satisfaction and workflow efficiency (Tierney 2025)	Observational: reduced documentation and total EHR time (Preiksaitis 2026). Generally accurate documentation (Williams 2025). Positive physician perceptions (Shy 2025; Alcázar-Peral 2026; Marquis 2026) Prospective: positive physician perceptions (Webb 2026) Simulation: faster than dictation (Lipinski 2026)	Hallucinations and omissions reported; continued evaluation of patient outcomes and documentation quality required; few controlled ED studies
	Telehospitalist (*n* = 10; ED = 3)	Remote overnight physician coverage for ED admissions	Implementation studies: feasibility, scalability, and sustainability (Gutierrez 2021; Becker 2021; Hron 2020; Papo 2025; Papo 2025). Reduced transfers (Kuperman 2018). No difference in clinical outcomes with traditional locum hospitalist care (Boltz 2019). High satisfaction (Gutierrez 2021; Kuperman 2018; Boltz 2019)	Implementation studies: nocturnal programs: positive patient and care team experience (JaKa 2020). Multisite feasibility (Nguyen 2026) Observational: optimized bed utilization and safe discharges (Koch 2026)	Limited ED-specific evidence; observational designs
Pillar 2: Expanding Workforce Capacity (*n* = 16; ED = 5)	Virtual nursing (*n* = 7; ED = 1)	Remote support for admissions, discharges, education, and observation	Scoping review: supports admission, discharge, and care coordination workflows; positive experience (Bhaloo 2025) Integrative review: clearly defined roles and education (Whalen 2025) Observational: no difference in clinical outcomes (Savitz 2023) Implementation reports and study: clearly defined roles (Nguyen 2024). Bedside nurse engagement (Welsh 2025, Schuelke 2019)	Observational: shorter wait times, fewer interruptions, fewer incomplete documentation fields (Khairat 2025)	Primarily descriptive evidence; limited ED-specific evidence; bedside nurse engagement critical
	Continuous RPM (*n* = 9; ED = 4)	Wearable vital sign surveillance for boarding ED patients	SR/MA: deployment feasibility and measurement validity (Leenen 2020). No differences in major events or complications, ICU admission, rapid response activation, LOS; varied mortality findings (Bowles 2024; Sun 2020; Areia 2021) Implementation report: multisite scalability (Nguyen 2026)	Feasibility study: deployment feasible and acceptable (Miller 2021) Validation study: RPM measurements strongly correlated with manual measurements (Garbern 2019) Predictive study: prediction of septic shock (Choi 2022) Prospective study: earlier detection of deterioration (Reed 2021)	No conclusive clinical outcome benefit; alarm optimization affects effectiveness; ED evidence limited to feasibility, validation, and predictive modeling
Pillar 3: Extending Continuity of Care (*n* = 12; ED = 7)	Virtual specialty consultation (*n* = 5; ED = 3)	Remote subspecialist access (stroke, psychiatry, ICU)	SR/MA, tele-ICU: lower ICU mortality, hospital mortality, and ICU LOS (Chen 2018) SR, telepsychiatry: implementation feasibility (Schulte 2026)	SR/MA, telestroke: no difference in safety and clinical outcomes (Mohamed 2024) Scoping review, telepsychiatry: feasible, may reduce boarding (Shalev 2024) Observational, tele-ICU: lower in-hospital mortality among patients admitted to ICU (Kadar 2019)	Substantial program heterogeneity; telepsychiatry effectiveness evidence limited
	Postdischarge transitional care (*n* = 7; ED = 4)	Virtual follow-up after ED discharge to reduce unplanned returns	SR/MA: reduced readmissions (Becker 2021; Tyler 2023; Sakashita 2025). Effects varied by intervention complexity (Tyler 2023)	Scoping review: comprehension-focused interventions most effective (Gorlick 2025) SR: Telephone follow-up alone ineffective (van Loon-van Gaalen 2021) Observational: structured programs reduced ED revisits (Light 2025; Lee 2025)	Heterogeneous transitional care interventions; most evidence posthospitalization rather than post-ED

*AI*, artificial intelligence; *ED*, emergency department; *EHR*, electronic health record; *ICU*, intensive care unit; *LOS*, length of stay; *M*, million; *MA*, meta-analysis; *RCT*, randomized controlled trial; *RPM*, remote patient monitoring; *SR*, systematic review.

**Table 2:** Proposed prioritization of digital health technologies for resource-limited systems.

Technology	Tier	Infrastructure Burden	Rationale
Virtual specialty consultation	Foundational	Moderate (contracted service, video capability, EHR integration)	Supplies subspecialty expertise not available on site; without it, patients are transferred or go without. Strongest ED-specific evidence base of the six, particularly for telestroke. Specific services should be selected based on local gaps in care.
Postdischarge transitional care	Foundational	Low; digital layer optional (existing staffing, automated messaging)	Supplies continuity of care that does not otherwise exist after ED discharge. Technology scales the model rather than creating the benefit; telephone follow-up alone was ineffective, whereas structured, comprehension-focused programs reduced revisits, which suggests that intervention design matters more than modality. Automation extends reach at low marginal cost as volume grows.
Telehospitalist	Enhancing (conditional)	Moderate (contracted or shared staffing model, standardized admitting workflows)	Conditional on local staffing structure: applicable to EDs with limited onsite hospitalist coverage, and of little value where coverage already exists. Evidence supports operational feasibility, particularly during overnight hours when staffing constraints may be greatest.
Ambient AI documentation	Enhancing	Moderate (recurring per-clinician licensing; no capital-free entry point)	Documentation occurs with or without it; the benefit accrues to clinician burden rather than clinical capability. Cost is recurring rather than one-time, which is harder to sustain under constrained operating budgets. May reduce documentation time and clinician burden, although clinical outcome effects are uncertain and ongoing clinician oversight is required given the risk of hallucinations and omissions.
Virtual nursing	Enhancing	High (dedicated remote nursing coverage; economical only across multiple units or sites)	Redistributes nursing work that occurs regardless. May improve nursing workflow and documentation completeness, but ED-specific evidence is limited to a single multicenter study. The staffing model depends on scale that smaller systems may not have.
Continuous RPM	Enhancing	High (wearable devices, EHR integration, centralized alarm management)	Monitoring occurs through manual vital signs; continuous capture increases frequency rather than adding a capability. Clinical outcome benefits remain inconclusive, and implementation is the most resource-intensive of the six. Reasonable to defer.

Foundational technologies (must-have) supply a clinical or operational capability the ED otherwise lacks; enhancing technologies (nice-to-have) improve a capability already present. Infrastructure burden reflects capital, licensing, and staffing requirements for initial implementation. Priority classifications are proposed and should be adapted to local staffing gaps, infrastructure, resources, and needs.

*AI*, artificial intelligence; *ED*, emergency department; *EHR*, electronic health record; *RPM*, remote patient monitoring.
